# Combining Lovastatin and Minocycline for the Treatment of Fragile X Syndrome: Results From the LovaMiX Clinical Trial

**DOI:** 10.3389/fpsyt.2021.762967

**Published:** 2022-01-04

**Authors:** Camille Champigny, Florence Morin-Parent, Laurence Bellehumeur-Lefebvre, Artuela Çaku, Jean-François Lepage, François Corbin

**Affiliations:** ^1^Department of Biochemistry and Functional Genomics, Faculty of Medicine and Health Sciences, Université de Sherbrooke, Sherbrooke, QC, Canada; ^2^Centre de Recherche du CHUS (CRCHUS), Sherbrooke, QC, Canada; ^3^Faculty of Medicine and Health Sciences, Department of Pediatrics, Université de Sherbrooke, Sherbrooke, QC, Canada

**Keywords:** fragile X syndrome, clinical trial, lovastatin, minocycline, intellectual disability, autism spectrum disorder (ASD), dual treatment

## Abstract

**Background:** Limited success of previous clinical trials for Fragile X syndrome (FXS) has led researchers to consider combining different drugs to correct the pleiotropic consequences caused by the absence of the Fragile X mental retardation protein (FMRP). Here, we report the results of the LovaMiX clinical trial, the first trial for FXS combining two disease-modifying drugs, lovastatin, and minocycline, which have both shown positive effects when used independently.

**Aim:** The main goals of the study were to assess the safety and efficacy of a treatment combining lovastatin and minocycline for patients with FXS.

**Design:** Pilot Phase II open-label clinical trial. Patients with a molecular diagnostic of FXS were first randomized to receive, in two-step titration either lovastatin or minocycline for 8 weeks, followed by dual treatment with lovastatin 40 mg and minocycline 100 mg for 2 weeks. Clinical assessments were performed at the beginning, after 8 weeks of monotherapy, and at week 20 (12 weeks of combined therapy).

**Outcome Measures:** The primary outcome measure was the Aberrant Behavior Checklist-Community (ABC-C) global score. Secondary outcome measures included subscales of the FXS specific ABC-C (ABC-C_FX_), the Anxiety, Depression, and Mood Scale (ADAMS), the Social Responsiveness Scale (SRS), the Behavior Rating Inventory of Executive Functions (BRIEF), and the Vineland Adaptive Behavior Scale second edition (VABS-II).

**Results:** Twenty-one individuals out of 22 completed the trial. There were no serious adverse events related to the use of either drugs alone or in combination, suggesting good tolerability and safety profile of the combined therapy. Significant improvement was noted on the primary outcome measure with a 40% decrease on ABC-C global score with the combined therapy. Several outcome measures also showed significance.

**Conclusion:** The combination of lovastatin and minocycline is safe in patients for FXS individuals and appears to improve several elements of the behavior. These results set the stage for a larger, placebo-controlled double-blind clinical trial to confirm the beneficial effects of the combined therapy.

## Introduction

FXS is an X-linked neurodevelopmental disorder caused by a CGG trinucleotide repeat expansion at the 5′ untranslated region of the FMR1 gene leading to its methylation and its consequent silencing. This results in reduced or absent expression of the FMRP, which is essential for proper brain development and synaptic functioning (1, 2). Clinically, FXS is characterized by moderate to severe intellectual disability in males, often accompanied by aggressivity and social avoidance, while females generally display a milder and broader cognitive phenotype. The neuropsychiatric profile of FXS includes anxiety, autism spectrum disorder (ASD) and attention deficit and/or hyperactivity disorder (ADHD). Current treatments are mostly symptomatic with limited efficacy. The latter includes antidepressants, stimulants, alpha2-agonists, and antipsychotics (3). There is a crucial need to better understand FXS core pathophysiology in order to find disease-modifying interventions capable of changing the natural trajectory of FXS and significantly reduce family burden (4).

In FXS, lack of FMRP leads to the hyper-phosphorylation of extracellular signal-regulated kinase (ERK), notably in mouse brain (5), human post-mortem brain (6, 7), and human platelets (8). Several lines of evidence suggest that reducing ERK activity reverts core features of the neurological phenotype of FXS animal models, including increased protein synthesis activity (9), cortical hyperexcitability and susceptibility to audiogenic seizures (10). These abnormalities, robustly found in preclinical models, recapitulate the enhanced risk of seizures of FXS patients and the presence of cortical hyperexcitability (11–13). Moreover, a clinical trial using lovastatin, a lipid-lowering drug that inhibits the mevalonate pathway and consequently lowers ERK phosphorylation (14), has been shown to improve the behavior of individuals with FXS aged 10 to 40 years in the context of a 3-month open-label trial (15). Interestingly, the observed decrease in ABC-C global score was somehow linked to the decrease of ERK phosphorylation in platelets, suggesting a mechanistic relationship between this pathway and behavioral outcome (8). More recently, a randomized 20-week placebo-controlled trial with lovastatin (10–40 mg/day) in 30 FXS participants (10–17 years old) was carried out introducing a parent-implemented language intervention (PILI) as primary outcome measure (16). Improved in PILI was reported in both groups during the trial without significant changes in ABC-C global scores.

In parallel to ERK phosphorylation, lack of FMRP also leads to increased matrix metalloproteinase-9 (MMP-9) activity, which has been ubiquitously described in mouse brain (17), human post-mortem brain (18, 19), and human plasma (20). Minocycline is a tetracycline antibiotic, an inhibitor of MMP-9 that showed its potency in the Fmr1 ko mouse correcting dendritic spine abnormalities (17), synaptic structures (21) while improving behavioral phenotypes (17, 22). In FXS participants, 8-weeks of treatment with minocycline was shown to improve behavior during an open-label clinical trial (23). The treatment was well-tolerated although seroconversion was reported in two subjects described as increased Anti-Nuclear Antibodies (ANA) titer. More recently, a double-blind placebo-controlled trial found that minocycline given for 3 months significantly improved the Clinical Global Impressions Scale-Improvement (CGI-I) score in children with FXS (ages 3.5–16 years) (24). Interestingly, in this double-blind trial, minocycline was shown to reduce plasmatic MMP-9 activity (20) and to improve their habituation to sound (25). Minocycline is therefore a promising drug that could correct core features of FXS pathophysiology.

Several other drugs have been tested over the years but none of them has shown clear efficacy in FXS placebo-controlled clinical trials (26). For this reason, and since the absence of FMRP has pleiotropic effects, a multi-targeted approach, combining drugs impacting different signaling pathways, could be more efficient to compensate for the absence of FMRP (27). However, there is a potential risk to target distinct receptor leading to a common mechanism such as blocking mGluR receptors and stimulate GABA in order to reduce cortical hyper-excitability (28). Since lovastatin and minocycline target clear distinct pathways, and have a very high security profile while being metabolized very differently, we believe that this synergistic drug regimen will have a higher positive effect on behavior in FXS participants, making them prime candidates for such an endeavor.

Here, we conducted an open-label, clinical trial combining lovastatin and minocycline in adolescents and adults with FXS to assess the safety and effectiveness of a combined therapy. We hypothesized that the combination of lovastatin and minocycline, each targeting distinct pathways, may have synergistic effects on cognition and behavior in individuals with FXS while not having added adverse effects.

## Materials and Methods

### Study Design

The study was an open-label, single center, clinical trial designed to evaluate the safety and (to some extent) the efficacy of a dual treatment lovastatin/minocycline in adolescents and adults with FXS. The study took place at the *Centre de Recherche du CHUS* (CRCHUS), Sherbrooke, Quebec, Canada. The enrollment period was from June 2016 to May 2017 with the last participant completing the trial in November 2017. Since dual therapy brings cumulated risk of adverse effects that could arise from either lovastatin, minocycline alone or in combination, direct exposition to both treatment at the beginning of the trial was avoided in accordance with Ethics Board recommendations, particularly in the context of a substitute consent. Prior exposition to either drugs as monotherapy for 8 weeks before 12 weeks with dual therapy was chosen to reassure participants and caregivers while facilitating monitoring of arising adverse effect's origin. This unique design also allowed the direct comparison between lovastatin and minocycline during the monotherapy period. The study was approved by the Ethics and Research Board of the CRCHUS and Health Canada being conformed to the Declaration of Helsinki.

### Participants Eligibility and Regimen Allocation

Males and females with: (i) a molecular diagnosis of FXS, (ii) aged between 13 and 45 years, (iii) with an age-adjusted Wechsler Intelligence Scale score (Full Scale IQ) <70, (iv) who has a caregiver that spends at least 6 h per day with the participant and attends all visits, were eligible to participate. Exclusion criteria included the following: (i) >3 psychoactive drugs, (ii) changes in treatment regimen in the last 3 months, (iii) severe or unstable disease, (iv) pregnancy, (v) history of sustained muscle enzymes elevation and/or muscle pain, (vi) history of liver, kidney disease or systemic lupus erythematosus, (vii) concomitant drugs being metabolized by cytochrome P450 3A4.

The study was presented in detail to caregivers (being also legal representatives) and explained to participants with the help of a picture diagram. Written informed consents from legal representatives along with verbal assents from participants were obtained. Eligible participants were then assigned to lovastatin or minocycline group by the pharmacy of the CRCHUS on a 1:1 basis to treatment arms, minimizing differences in covariates (gender and age). Participants in the lovastatin group took lovastatin 20 mg during 4 weeks and 40 mg for 4 weeks, while participants in the minocycline group started with minocycline 50 mg and then 100 mg daily. Then, both groups received combined treatment of lovastatin 40 mg and minocycline 100 mg for 12 weeks. Visits at the research center were scheduled at baseline, week 8, 12, and 20 and phone calls monitoring were at week 4 and week 24 (4 weeks after completion of the trial).

### Medication

Lovastatin tablets (20 and 40 mg) and minocycline capsules (50 and 100 mg) were obtained from Apotex (Toronto, Ontario, Canada). Caregivers were instructed that participants should take their medication orally every morning. If a participant had difficulty swallowing pills, we instructed caregivers that the minocycline capsules can be opened, and lovastatin tablets crushed if needed. Furthermore, caregivers were instructed that participants should avoid eating grapefruits as well as multivitamins or anti-acids during the study. To monitor compliance, subject diaries were given to caregivers and remaining tablets or capsules were counted (compliance = number of tablets taken/number of days between visits^*^100%).

### Baseline Evaluation

Medical history, medication, and demographic information were collected at baseline. The designated caregiver filled out the French version of the Social Communication Questionnaire (SCQ). Full-scale IQ (FSIQ) of participants was assessed with the French version either of the Wechsler Intelligence Scale for Children Fourth Edition (WISC-IV) or the Wechsler Adult Intelligence Scale Third Edition (WAIS-III) by a qualified neuropsychologist. The treating specialist (FC) filled out the Clinical Global Impressions Scale-Severity (CGI-S) which ranges from 1 (“Normal, not at all ill”) to 7 (“Among the most extremely ill patients”) (29). Molecular diagnosis was assessed by Southern Blot and PCR-based genotyping and non-classic mutations were detected by array comparative genomic hybridization. Platelet content in FMRP was measured by immunoblots as previously described (30).

### Outcome Measures

The primary outcome was the global score of the ABC-C (31, 32). Secondary outcomes included each of the FXS ABC-C subscales (ABC-C_FX_) subscales (33), the ADAMS, the BRIEF, the SRS, the VABS-II, and the Test of Attentional Performance for Children (KiTAP) (34). With the exception of the KITAP, all questionnaires were filled out by the same caregiver of the participant, at baseline, week 8, 12, and 20 according to the behavior of the participant in the previous 2 weeks.

### Safety

Vital signs, weight measurements, and physical examination were carried out at each visit. Blood samples were drawn from non-fasting participants at each visit to performed biochemical tests. Creatinine, creatine kinase (CK), alkaline phosphatase (ALP), bilirubin, alanine aminotransferase (ALT), total cholesterol (TC), high-density lipoprotein cholesterol (HDL-C) were measured by enzymatic/spectrophotometric method (Roche Diagnostics^®^ Modular P700) and apolipoprotein B (ApoB) by immunoturbidimetric assay (Roche Diagnostics^®^ Cobas e501). Anti-nuclear antibodies (ANA) titer was determined by immunofluorescence. Non-HDL-Cholesterol (Non-HDL-C) was calculated as the difference between TC and HDL-C. Adverse events (AE) were systematically investigated with both open and close-ended questions at each visit and each scheduled phone call. Close-ended questions included those more frequently associated to lovastatin (muscle and joint pain) and minocycline treatment (teeth or skin coloration). The Common Terminology Criteria for Adverse Events (CTCAE) Version 4.0 (35) was applied to report AE and their severity, ranging from 1 (“Mild”) to 5 (“Death related AE”). A difference in ALP, creatinine, bilirubin, ALT, CK, CT, HDL, non-HDL-C, or ApoB compared to the baseline was included in the qualitative description of AE. The Liverpool causality tool was used to determine drug causality of adverse events on a 4-point scale, ranging from “Unlikely” to “Definite” (36).

### Statistical Analysis

A power analysis based on previous clinical trial in FXS using ABC-C global score lessening as the primary outcome recommended a sample size of 11 for each group would have at least 80% power to detect a difference in means of 19, assuming a standard deviation of differences of 19.3, using a paired *t*-test with an alpha level of 0.05 (two-sided). Data were analyzed on an intention-to-treat method, and the normality of data distribution was assessed with the Shapiro-Wilk test before applying paired Wilcoxon or paired Student's *t*-test accordingly. Following the assessment of normality with the Shapiro-Wilk test (significant at alpha 5%), differences in participants' baseline information were assessed with Wilcoxon or *t*-test for continuous variables, and with Fisher's exact test for categorical variables. Similarly, normality was assessed before applying paired Wilcoxon or paired Student's *t*-test for the differences in the primary (ABC-C_FX_ Global Score) and all secondary outcomes. To increase sensitivity for the BRIEF questionnaire, questions 21, 24, 38, and 72 and the Plan/Organize subdomain were excluded from the analysis due to too many questions being non-applicable to FXS patients. For all questionnaires, missing answers were treated according to the provider's booklet. If an answer was missing on a questionnaire for a specific participant, the specific related question was eliminated in the same way on all the other questionnaires of the participant. Adverse events were assessed with descriptive statistics. Due to the exploratory nature of the trial, we report the uncorrected *p*-values. Statistical analyses were performed with R software version 3.3.3 and GraphPad Prism version 9.2.0.

## Results

### Participants

**Figure 1:**
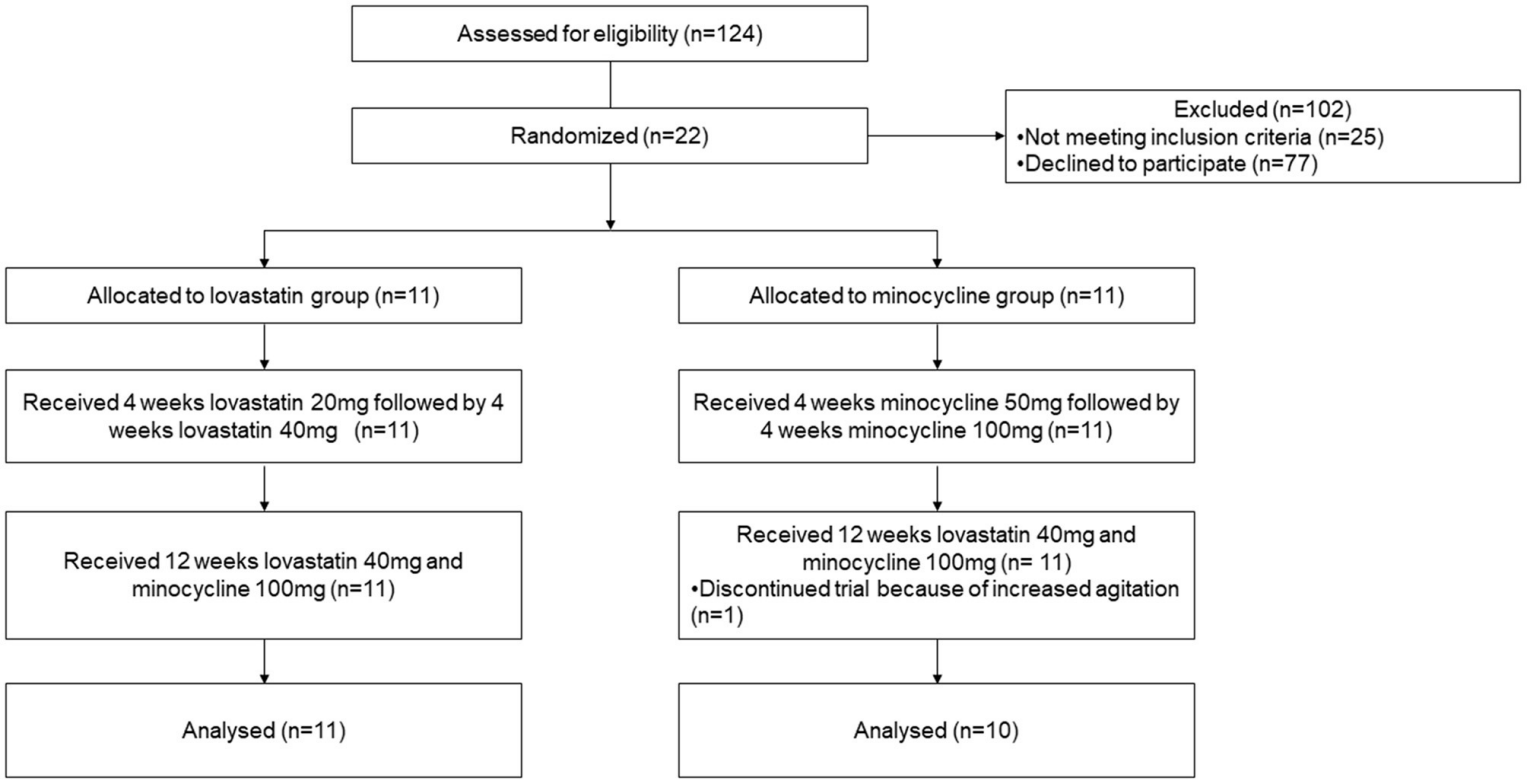
CONsolidated Standards of Reporting Trials (CONSORT) flow diagram of subject disposition.

One hundred and twenty-four patients were contacted either directly or by e-mail to participate in the study (Figure 1). Of these, 77 patients declined to participate and 25 were not eligible. Recruited participants were randomly allocated to either lovastatin or minocycline group (11 per group). Only one participant out of 22 did not complete the trial. The latter was withdrawn from the study by the caregiver because of increased agitation while on the combined treatment. No statistically significant difference was found between baseline demographic information collected from both groups (Table 1). Of note, one female who had a deletion in the FMR1 gene instead of the classic mutation was included in the study. Noticeably, more than half of our participants were not taking any psychoactive medication before entering the study.

**Table 1:** Baseline characteristics of participants.

**Baseline characteristic**	**Lovastatin group (*n* = 11)**	**Minocycline group (*n* = 11)**
Age	26 (19.5–26.5)	22 (19.5–25.5)
Gender—no. (%)		
Male	10 (90.91%)	9 (81.82%)
Female	1 (9.09%)	2 (18.18%)
Ethnicity—no. (%)		
Caucasian	10 (90.91%)	11 (100%)
African	1 (9.09%)	0 (0%)
Mutation type—no. (%)		
Full mutation	10 (90.91%)	10 (90.91%)
Male mosaic	1 (9.09%)	0 (0%)
Deletion	0 (0%)	1 (9.09%)
FMRP^†^ (pg/10^6^ platelets)	0 (0–8.2)	0 (0–5.5)
Positive ANA*^‡^*–no. (%)	1 (0.09%)	4 (36.36%)
FSIQ	45 (41–51.5)	48 (42–56)
SCQ score	14 (11–16.5)	15 (8–16.5)
CGI-S median (range)	4 (4–5)	4 (4–5)
Living Setting—no. (%)		
Home with family	11 (100%)	11 (100%)
Number of concomitant psychoactive medication—no. participant (%)		
None	7 (63.64%)	6 (54.55%)
One	3 (27.27%)	2 (18.18%)
Two	1 (9.09%)	0 (0%)
Three	0 (0%)	3 (27.27%)
Type of medication—no. (%)		
Antidepressant	2 (40%)	4 (36.36%)
Stimulant	2 (40%)	1 (9.09%)
Alpha2-adrenergic agonist	0 (0%)	1 (9.09%)
Antipsychotic	1 (20%)	5 (45.45%)

*Data are presented as median (interquartile range) unless otherwise specified. ANA, anti-nuclear antibodies; FSIQ, full scale intellectual quotient; SCQ, social communication questionnaire; CGI-S, clinical global impressions scale-severity*.

*Quantified by western blot*.

‡ *ut-off titer at 1:80*.

### Compliance, Safety, and Adverse Events

Overall, mean compliance to medication for mono- and combined therapy was higher than 97% (Table 2). Adverse events occurring during the trial were of mild or moderate severity except for one participant who had a severe increase in ALT (>5.0 upper limit normal (ULN)) during the combined treatment period (Table 2). For all AE, drug-related causality ranged from unlikely to probable with none being definite. More adverse events occurred during the combined treatment phase than during monotherapy. Intriguingly, headache was again reported in few participants during lovastatin treatment as noted previously (15). Regarding biochemical tests, CK elevation was the most reported AE after 20 weeks of treatment, a well-known effect of statins. However, there was no myalgia reported by the participants. As expected, lovastatin lowered mean TC, HDL-C, ApoB of the lovastatin but minocycline groups (Table 3). In order to carefully monitor seroconversion, ANA titer was determined at baseline and during the course of the study. Unexpectedly, a few participants had already a positive (ANA) at baseline (Table 1) but no seroconversion or significant increase in ANA occurred during the trial (data not shown).

**Table 2:** Adverse events (AE).

**Adverse events**	**Number of subjects (*n* = 22)**	**Number of events**	**Treatment period**			Grade of adverse event^*^			**Drug-related causality** ^**†**^		
			**Lovastatin (*n* = 11)**	**Minocycline (*n* = 11)**	**Bitherapy (*n* = 22)**	**Mild**	**Moderate**	**Severe**	**Unlikely**	**Possible**	**Probable**
Aggressivity	1	1			✓		✓				✓
Agitation	4	4	✓		✓✓✓	✓✓	✓✓		✓	✓✓	✓
ALP increase	1	1			✓	✓			✓		
ALT increase	2	2	✓		✓	✓		✓			✓✓
Anal pruritus	1	1			✓	✓					✓
Anorexia	4	4		✓	✓✓✓	✓✓✓✓				✓	✓✓✓
Arthralgia	1	1	✓			✓			✓		
Back pain	1	1	✓			✓			✓		
Blood bilirubin increase	1	1			✓		✓*^**‡**^*				✓
Cough	1	1	✓			✓			✓		
CK increase	5	5			✓✓✓✓✓	✓✓✓✓✓					✓✓✓✓✓
Diarrhea	3	3		✓	✓✓	✓✓✓				✓	✓✓
Dizziness	1	1		✓		✓				✓	
Dysmenorrhea	1	1			✓	✓			✓		
Dyspepsia	1	1			✓		✓			✓	
Fatigue	2	3		✓✓	✓	✓✓✓				✓✓✓	
Gastroenteritis	1	1	✓			✓			✓		
Headache	4	4	✓✓✓	✓		✓✓✓✓			✓✓✓	✓	
Hypersomnia	2	2		✓	✓	✓	✓			✓✓	
Neck pain	1	1			✓		✓		✓		
Pain in extremity	1	1	✓			✓			✓		
Palpitations	1	1		✓		✓				✓	
Panic attack	1	1		✓		✓				✓	
Pharyngitis	1	1			✓		✓		✓		
URTI	6	6	✓✓✓		✓✓✓		✓✓✓✓✓		✓✓✓✓✓		
Vomiting	2	2	✓		✓	✓	✓		✓	✓	
URTI	6	6	✓✓✓		✓✓✓		✓✓✓✓✓		✓✓✓✓✓		
CK increase	5	5			✓✓✓✓✓	✓✓✓✓✓					✓✓✓✓✓
Agitation	4	4	✓		✓✓✓	✓✓	✓✓		✓	✓✓	✓
Anorexia	4	4		✓	✓✓✓	✓✓✓✓				✓	✓✓✓
Headache	4	4	✓✓✓	✓		✓✓✓✓			✓✓✓	✓	
Diarrhea	3	3		✓	✓✓	✓✓✓				✓	✓✓
Fatigue	2	3		✓✓	✓	✓✓✓				✓✓✓	
ALT increase	2	2	✓		✓	✓		✓			✓✓
Hypersomnia	2	2		✓	✓	✓	✓			✓✓	
Vomiting	2	2	✓		✓	✓	✓		✓	✓	
Aggressivity	1	1			✓		✓				✓
ALP increase	1	1			✓	✓			✓		
Anal pruritus	1	1			✓	✓					✓
Arthralgia	1	1	✓			✓			✓		
Back pain	1	1	✓			✓			✓		
Blood bilirubin increase	1	1			✓		✓*^**‡**^*				✓
Cough	1	1	✓			✓			✓		
Dizziness	1	1		✓		✓				✓	
Dysmenorrhea	1	1			✓	✓			✓		
Dyspepsia	1	1			✓		✓			✓	
Gastroenteritis	1	1	✓			✓			✓		
Neck pain	1	1			✓		✓		✓		
Pain in extremity	1	1	✓			✓			✓		
Palpitations	1	1		✓		✓				✓	
Panic attack	1	1		✓		✓				✓	
Pharyngitis	1	1			✓		✓		✓		

*ALP, alkaline phosphatase; ALT, alanine aminotransferase; CK, creatine kinase; URTI, upper respiratory tract infection*.

* *The Common Terminology Criteria for Adverse Events (CTCAE) Version 4.0 were used to describe AE. Severity ranges from 1 (“Mild”) to 5 (“Death related AE”). ✓ represents each event. For biochemical values, the following ranges were used: mild (ALP > upper limit of normal (ULN) 2.5xULN, ALT > ULN−3.0xULN, bilirubin > ULN−1.5xULN, CK > ULN−2.5xULN), moderate (ALP > 2.5xULN−5.0xULN, ALT > 3.0xULN−5.0xULN, bilirubin > 1.5xULN−3.0xULN, CK > 2.5xULN−5.0xULN), and severe (ALP > 5.0xULN−20.0xULN, ALT > 5.0ULN−20.0xULN, bilirubin > 3.0ULN−10.0xULN, CK > 5.0xULN−10.0xULN). Only participants who increased in categories compared to baseline for the biochemical values were included as adverse events*.

† *Drug-related causality was measured by the Liverpool causality tool*.

‡ *aseline bilirubin value for this participant was of mild intensity*.

**Table 3:** Biochemical measurements.

**Biochemical variables**	Reference intervals^*^	**Lovastatin group**			**Minocycline group**		
		**Baseline (*n* =11)**	**8 weeks (*n* = 10)**	**20 weeks (*n* = 11)**	**Baseline (*n* = 11)**	**8 weeks (*n* = 11)**	**20 weeks (*n* = 10)**
CK	≤ 150 IU/L	84.27 ± 27.24	92.5 ± 30.27	105.27 ± 49	64.18 ± 28.91	70.82 ± 26.78	67.90 ± 21.86
Creatinine	37–110 μm/L	65.64 ± 8.97	64.8 ± 10.13	69.55 ± 7.98	58.09 ± 14.85	58.73 ± 15.26	60.20 ± 16.27
Lipid profile							
TC	3.2–5.48 mmol/L	3.58 ± 0.74	2.83 ± 0.61	2.92 ± 0.55	3.71 ± 0.59	3.61 ± 0.82	2.92 ± 0.48^†^
HDL-C	0.69–1.81 mmol/L	1.01 ± 0.16	1.06 ± 0.18	1.07 ± 0.22	1.02 ± 0.19	1.04 ± 0.24	1.08 ± 0.31
Non-HDL-C		2.57 ± 0.77	1.77 ± 0.68	1.85 ± 0.68	2.69 ± 0.69	2.57 ± 0.89	1.85 ± 0.64^†^
Apo B	0.6–1.6 g/L	0.77 ± 0.2	0.59 ± 0.17	0.63 ± 0.17	0.82 ± 0.23	0.78 ± 0.24	0.66 ± 0.19^†^
Liver profile							
ALP	35–485 IU/L	97.91 ± 74.44	96.9 ± 73.04	97 ± 67.96	119 ± 94	125.6 ± 103.2	127.2 ± 94.9
ALT	≤ 55 IU/L	26 ± 14.46	28.2 ± 14.53	26.73 ± 11.52	23.91 ± 17.52	31.09 ± 25.35	71.8 ± 125.4^†^
Bilirubin	2.8–17 μm/L	12.42 ± 8.59	13.78 ± 7.25	15.2 ± 14.19	8.89 ± 4.92	9.15 ± 5.66	11.65 ± 11.51

*Data are presented as mean ± SD. CK, creatine kinase; TC, total cholesterol; HDL-C, high-density lipoprotein cholesterol; Non-HDL-C, non-high-density lipoprotein cholesterol; Apo B, apolipoprotein B; ALP, alkaline phosphatase; ALT, alanine aminotransferase*.

* *Reference intervals are in accordance with Roche Diagnostics^®^ technical sheets*.

† *p ≤ 0.01*.

### Efficacy Analyses

We observed a 40% improvement in the ABC-C Global Score, our primary outcome, after 20 weeks of treatment with both drugs when all participants of each arm are combined (Table 4). Several subscales were also improved either in ABC-C_FX_, ADAMS, SRS, and BRIEF. There was also improvement in some VABS scores and in “errors with distractor” task of the KiTAP (Supplementary Table 1). When each group was taken separately, much less outcome measures remained statistically significant, an effect mostly attributable to smaller sample size. Nevertheless, the lovastatin but not the minocycline group showed improvement in SRS total score (Table 4), an effect already significant after 8 weeks of treatment with lovastatin alone (Supplementary Table 2).

**Table 4:** Outcome measures 0–20 weeks.

**Endpoints**	**Lovastatin group (*****n*** **=** **11)**			**Minocycline group (*****n*** **=** **10)**			**Both groups (*****n*** **=** **21)**		
	**Baseline**	**20 weeks**	***p***	**Baseline**	**20 weeks**	***p***	**Baseline**	**20 weeks**	***p***
ABC-C_FX_									
Global score	48 (35–58)	33 (24–40)	0.054	41 (26–65)	24 (16–53)	0.143	48 (27–62)	29 (16–45)	**0.004**
Irritability	8 (3–10)	3 (1–9)	0.412	6.5 (1–14)	3 (1–9)	0.617	8 (3–11)	3 (1–10)	0.306
Lethargy	8 (6–14)	7 (3–8)	0.129	10.5 (6–14)	5.5 (1–11)	**0.009**	10 (5–14)	6 (2–9)	**0.005**
Stereotypy	6 (4–10)	5 (4–6)	0.191	5 (3–6)	4.5 (2–5)	0.375	5 (3–10)	5 (3–5)	0.111
Hyperactivity	7 (4–9)	3 (2–7)	**0.014**	5.5 (4–10)	4 (2–7)	0.262	6 (4–9)	3 (2–7)	**0.006**
Inappropriate speech	5 (4–7)	5 (3–6)	0.176	6.5 (4–8)	3.5 (2–8)	0.103	5 (4–5)	4 (2–4)	**0.028**
Social avoidance	8 (7–11)	5 (4–8)	**0.027**	5.5 (4–8)	4.5 (2–8)	0.406	7 (5–10)	5 (4–8)	**0.020**
ADAMS									
Total score	28 (17–40)	23 (16–29)	**0.043**	30 (14–49)	16 (10–37)	0.082	28 (17–40)	19 (12–29)	**0.008**
Manic/hyperactive behavior	5 (3–6)	3 (3–6)	0.169	6 (3–9)	4.5 (2–6)	0.152	5 (3–7)	4 (2–6)	**0.047**
Depressed mood	3 (1–4)	1 (1–4)	0.177	4.5 (1–10)	3.5 (1–6)	0.586	3 (1–6)	2 (1–4)	0.202
Social avoidance	11 (8–15)	8 (6–11)	0.076	8 (6–17)	5.5 (4–11)	0.056	9 (6–15)	7 (5–12)	**0.007**
General anxiety	5 (3–13)	4 (3–8)	0.063	6.5 (4–10)	3 (0–8)	0.078	6 (3–11)	3 (1–8)	**0.008**
Obsessive/compulsive behavior	2 (2–3)	2 (1–4)	0.440	4 (1–5)	1 (0–3)	0.172	2 (1–4)	2 (0–3)	0.074
BRIEF									
Inhibit	24 (20–28)	21 (19–26)	**0.005**	22.5 (19–27)	20.5 (17–23)	**0.021**	23 (20–27)	21 (17–23)	**<0.001**
Shift	23 (20–25)	19 (18–21)	**0.010**	20 (18–23)	18 (17–21)	**0.042**	21 (19–24)	19 (17–21)	**0.001**
Emotion control	17 (15–20)	15 (14–19)	**0.018**	14.5 (13–18)	13 (12–17)	0.513	16 (14–19)	15 (12–18)	**0.043**
Monitor	10 (9–12)	10 (9–11)	0.703	10.5 (8–13)	9 (7–12)	0.096	10 (8–12)	9 (8–11)	0.124
Working memory	24 (22–27)	23 (22–24)	0.092	21.5 (20–25)	21 (20–23)	0.181	22 (20–25)	22 (20–23)	**0.026**
Organize materials	10.5 (8–13)	9.5 (8–12)	0.943	9 (7–11)	9.5 (8–12)	0.052	9 (7–11)	9 (7–12)	0.366
Task completion	21 (18–22)	18 (16–22)	0.205	17.5 (15–19)	15.5 (13–18)	0.290	18 (15–19)	16 (13–18)	0.087
SRS									
Total raw score	163 (157–170)	149 (139–159)	**<0.001**	150 (130–174)	135 (131–149)	0.081	162 (138–171)	146 (133–159)	**<0.001**
Awareness	19 (18–21)	17 (17–21)	0.632	19 (18–20)	17 (16–19)	0.078	19 (18–21)	17 (16–19)	0.097
Cognition	30 (27–33)	27 (25–32)	**0.003**	30 (26–33)	26.5 (26–28)	0.305	30 (26–33)	27 (25–29)	**0.015**
Communication	52 (50–58)	49 (42–56)	**0.009**	45.5 (44–54)	44.5 (41–49)	0.274	51 (44–57)	47 (41–50)	**0.009**
Motivation	29 (27–31)	25 (23–29)	**0.048**	25 (23–29)	23.5 (22–27)	0.144	27 (24–30)	25 (22–28)	**0.011**
Mannerisms	32 (28–34)	27 (25–30)	**0.001**	29 (23–35)	24.5 (22–30)	0.130	30 (26–34)	26 (23–30)	**0.001**

*Data are presented as median (interquartile range). P value < 0,05 are in bold. ABC-C_FX_, aberrant behavior checklist-community adapted for FXS; ADAMS, anxiety, depression and mood scale; BRIEF-SR, behavior rating inventory of executive function-self-report version; SRS, social responsiveness scale*.

### Compared Efficacy Lovastatin/Minocycline Monotherapy

The distinctive design of our study allowed us to study not only the effect of the combined treatment but also to measure and compare the monotherapy effect of lovastatin and minocycline for 8 weeks. Although improvements were noted in most scales, they were rarely significant (Supplementary Table 1). The combination of small sample size (11) and a short duration of treatment (8 weeks) could explain the latter. Noteworthy, as mentioned previously, SRS seems clearly more affected by lovastatin treatment. Improvements in Inappropriate Speech (ABC-C_FX_) and ADAMS was observed for minocycline but not lovastatin.

## Discussion

FXS remains a neurodevelopmental condition resulting from various alterations in absence of FMRP. The use of several medications with additive effects may be the key to a successful disease-modifying treatment (37). To our knowledge, this is the first trial assessing the safety and efficacy of a combined disease-modifying add on treatment in FXS individuals. Lovastatin and minocycline target specific pathways affected by the absence of FMRP and have shown promising effect in previous clinical trials (15, 23). These were specifically chosen because they both have a very good long-term safety profile for either treating hypercholesterolemia or acne vulgaris, respectively (38).

### Security

In fact, as previously reported in monotherapy trials (15, 16, 23, 24), most adverse events following lovastatin or minocycline administration were mild, self-limited, and poorly related to treatment with the exception of CK elevation. A high percentage of participants had an initial positive ANA titer (22.7%), which is not clinically significant in absence of symptoms. More importantly, no seroconversion was observed even though half of participants were exposed to minocycline for 20 weeks. Although more adverse events occured during the combined therapy period, the treatment was very well-tolerated with only one participant not completing the study. Some characteristics of our trial may have contributed to minimize side effects. For instance, half of our participants were not taking any psychoactive medication on a regular basis limiting the bias of unknown drug interactions. Also, participants were adolescents or adults and there was less concern regarding permanent teeth discoloration (38). However, long-term safety of this specific combined therapy remains to be determined. In fact, the unknown long-term risk of a sustained low cholesterol level and seroconversion should be carefully considered.

### ABC-C Global Score and ABC-C_FX_ Subscales

Regarding our primary outcome, we obtained a similar reduction in the ABC-C global score with the bi-therapy as compared to our previous open label trial with lovastatin (15). However, no significant improvement was obtained in ABC-C_FX_ subscales with lovastatin monotherapy. The absence of improvement may be related to the combination of fewer participants (11 vs. 15) and a shorter period of treatment (8 vs. 12 weeks). Similarly, we did not observe a significant reduction in ABC-C global score with minocycline as reported in another Canadian center (23). However, our trial was performed with nearly half participants (11 vs. 20). Altogether, global score and almost all ABC-C_FX_ subscales (4 of 6) showed statistically significant improvement making this combined treatment a very promising one.

### Other Outcomes

Several questionnaires were tested as secondary outcomes during the course of the trial, the majority being greatly recommended in FXS clinical trial (32). Many of them such as ADAMS, SRS, and BRIEF were not administered in previous trials with either minocycline or lovastatin monotherapy, greatly limiting comparison. Yet, many subscales of these questionnaires were improved and limited our ability to identify a better outcome measure than ABC-C global score. Also, beside SRS who appeared more specific to lovastatin treatment, we were unable to determine if lovastatin and minocycline improve differently, specific traits highlighted in those questionnaires.

### Study Limitations

Our pilot trial had clear limitations. Owing to the intrinsic design of the trial (open-label), outcome measures are prone to the placebo effect and observer-expectancy bias. Also, the distinct contribution of the monotherapy and the dual therapy period on the overall effect of the 20 week treatment is difficult to delineate since the contribution of the placebo effect in each treatment period is undetermined. In fact, caregivers have clearly showed higher concerns with the addition of the second drug that could modulate the placebo effect during the dual therapy period. Nevertheless, additional exposure to lovastatin's monotherapy for 8 weeks may explain higher SRS improvement in the lovastatin group. Alternatively, the minocycline group had more participants taking 3 or more psychoactive medications that could lead to lesser SRS improvement. It remains unclear if starting both medications simultaneously for 12 weeks would have been as beneficial in terms of efficacy while being as safe for participants. Our short trial duration and greater age of participants make it easier to monitor adverse effects but less likely to obtain significant effects on behavior. Clearly, to determine the true benefit potential of this combined lovastatin-minocycline treatment, a placebo-controlled trial is warranted in younger individuals where the two drugs are taken at the very beginning of the trial.

This study, clearly showing the security of a combined treatment would certainly alleviate caregiver apprehension on adverse effects and facilitate recruitment for future trials using a combined treatment of lovastatin/minocycline. Our study also paves the way for future trials using other combined treatment that would better compensate for the absence of FMRP and improve the natural evolution of FXS individuals and alleviate families' burden.

## Data Availability Statement

The raw data supporting the conclusions of this article will be made available by the authors, without undue reservation.

## Ethics Statement

The studies involving human participants were reviewed and approved by Comité d'éthique de la recherche du CIUSSS de l'Estrie - CHUS. Written informed consent to participate in this study was provided by the participants' legal guardian/next of kin.

## Author Contributions

CC conducted the trial, helped with coordination, instructed participants to perform the KiTAP, recorded clinical data, performed data analysis, and drafted the manuscript. FM-P conducted the Vineland interviews and the IQ testing. LB-L performed data analysis and drafted the manuscript. AÇ performed data analysis and conducted medical assessments. J-FL participated in the design of the study. FC conceived the study, participated in the design of the study, conducted medical assessments, and oversaw the execution of the trial. All authors participated to the data statistical analysis, contributed to the article, and approved the submitted version.

## Funding

This work was funded by the FRAXA Research Foundation (FC) and the Fonds de la Recherche du Québec-Santé (J-FL). CC was supported by a graduate scholarship from the New Brunswick Health Research Foundation and FM-P by the Foundation du Grand Défi Pierre-Lavoie.

## Conflict of Interest

The authors declare that the research was conducted in the absence of any commercial or financial relationships that could be construed as a potential conflict of interest.

## Publisher's Note

All claims expressed in this article are solely those of the authors and do not necessarily represent those of their affiliated organizations, or those of the publisher, the editors and the reviewers. Any product that may be evaluated in this article, or claim that may be made by its manufacturer, is not guaranteed or endorsed by the publisher.
