# Supplementary material for: Combining Lovastatin and Minocycline for the Treatment of Fragile X Syndrome: Results From the LovaMiX Clinical Trial
Source: Front Psychiatry. 2022 Jan 4;12:762967. doi: 10.3389/fpsyt.2021.762967 (PMC8763805; doi:10.3389/fpsyt.2021.762967)
Supplement: Supplementary file 1 [file Data_Sheet_1.docx]

**Table S1. Additional outcome measures 0-20 weeks.**

| Endpoints | | | Lovastatin group (n=11) | | | Minocycline group (n=10) | | | Both groups (n=21) | | |  |
| --- | --- | --- | --- | --- | --- | --- | --- | --- | --- | --- | --- | --- |
|  |  |  | Baseline | 20 weeks | p | Baseline | 20 weeks | p | Baseline | 20 weeks | p |  |
| KiTAP | | |  |  |  |  |  |  |  |  |  |  |
|  | Alertness | |  |  |  |  |  |  |  |  |  |  |
|  |  | RT median | 417 (402-470) | 424 (411-587) | 1 | 452 (344-817) | 488 (432-520) | 0.63 | 417 (374-533) | 460 (418-560) | 0.31 |  |
|  |  | RT SD | 143 (127-412) | 243 (226-280) | 0.63 | 254 (160-431) | 274 (118-367) | 0.63 | 202 (127-494) | 258(187-330) | 0.69 |  |
|  | Distractibility | |  |  |  |  |  |  |  |  |  |  |
|  |  | Omissions with distractor | 3 (2-4) | 3 (2-6) | 0.20 | 2 (2-3) | 1 (1-4) | 1 | 2 (2-4) | 2.5 (1-5) | 1 |  |
|  |  | Omissions without distractor | 1 (0-2) | 1 (1-3) | 0.59 | 1 (0-3) | 2 (1-3) | 0.61 | 1 (0-3) | 1 (1-3) | 0.66 |  |
|  |  | Total omissions | 4 (3-6) | 4 (3-9) | 0.31 | 4 (2-6) | 4 (3-7) | 1 | 4 (2-6) | 4 (3-8) | 0.69 |  |
|  |  | Errors with distractor | 11 (6-14) | 4 (1-12) | 0.09 | 10 (8-11) | 4 (4-11) | 0.45 | 10 (6-12) | 4 (2-12) | **0.04** |  |
|  |  | Errors without distractor | 15 (2-17) | 5 (3-14) | 0.67 | 7 (2-11) | 5 (3-13) | 0.83 | 8 (2-16) | 5 (3-14) | 0.78 |  |
|  |  | Total errors | 27 (8-31) | 9 (4-25) | 0.26 | 15 (12-21) | 9 (7-25) | 0.78 | 16 (11-27) | 9 (5-26) | 0.16 |  |
|  | Flexibility | |  |  |  |  |  |  |  |  |  |  |
|  |  | RT median | 741  (636-1069) | 806  (680-1029) | **0.04** | 794  (649-1112) | 887  (641-1072) | 0.11 | 753  (646-1069) | 839  (656-1051) | 0.13 |  |
|  |  | RT SD | 419 (260-541) | 367 (232-441) | **0.01** | 403 (305-901) | 316 (214-382) | 0.15 | 405 (283-615) | 316 (223-434) | 0.07 |  |
|  |  | Errors | 12 (11-14) | 11 (11-13) | **0.05** | 11 (11-12) | 14 (13-15) | **0.03** | 11 (11-13) | 13 (11-14) | 0.09 |  |
|  | Inhibition | |  |  |  |  |  |  |  |  |  |  |
|  |  | RT median | 487 (362-556) | 501 (416-546) | 0.95 | 450 (401-546) | 418 (383-516) | 0.25 | 487 (398-556) | 458 (400-538) | 0.93 |  |
|  |  | RT SD | 137(110-185) | 176 (114-217) | 0.38 | 130 (92-206) | 91 (88-106) | 0.95 | 137 (103-190) | 120 (89-206) | 0.21 |  |
|  |  | Errors | 3 (2-4) | 1 (1-4) | 0.28 | 2 (1-5) | 4 (2-9) | 0.40 | 3 (2-4) | 2 (1-7) | 0.41 |  |
| VABS II | | |  |  |  |  |  |  |  |  |  |  |
|  | Sum of scores | | 139 (131-155) | 151 (127-205) | 0.18 | 167 (135-194) | 192 (129-214) | 0.22 | 140 (132-193) | 163 (127-210) | **0.05** |  |
|  | Receptive | | 33 (30-35) | 34 (30-36) | 0.18 | 30 (26-34) | 33 (30-34) | 0.71 | 31 (29-35) | 33 (30-36) | 0.32 |  |
|  | Expressive | | 93 (85-94) | 92 (88-99) | **0.05** | 83 (77-90) | 92 (88-96) | 0.09 | 90 (77-94) | 92 (87-99) | **0.01** |  |
|  | Written | | 21 (17-29) | 17 (16-32) | 1 | 16 (14-22) | 17.5 (16-24) | 0.23 | 19 (15-29) | 17 (16-32) | 0.74 |  |
|  | Personal | | 72 (70-75) | 73 (72-75) | 0.14 | 69 (66-72) | 70 (66-74) | 0.23 | 72 (67-74) | 73 (68-75) | **0.05** |  |
|  | Domestic | | 34 (30-45) | 39 (29-43) | 0.29 | 33 (28-39) | 34 (33-37) | 0.44 | 33 (29-39) | 37 (31-41) | 0.17 |  |
|  | Community | | 55 (49-63) | 57 (56-59) | 0.21 | 49 (47-51) | 58.5 (51-62) | 0.07 | 50 (49-60) | 57 (55-62) | **0.03** |  |
|  | Interpersonal | | 62 (52-67) | 60 (48-67) | 0.59 | 65 (60-66) | 63 (58-67) | 1 | 63 (57-67) | 62 (52-67) | 0.59 |  |
|  | Play and leisure time | | 52 (44-53) | 53 (42-55) | 0.62 | 51 (44-53) | 45.5 (44-48) | 0.46 | 52 (44-53) | 48 (44-54) | 0.33 |  |
|  | Coping skills | | 47 (39-55) | 51 (47-55) | 0.17 | 42 (39-47) | 49.5 (40-55) | 0.44 | 46 (39-49) | 51 (44-55) | 0.12 |  |
| Data are presented as median (interquartile range). CGI-I, Clinical Global Impressions Scale-Improvement;KiTAP, Test of Attentional Performance for Children; RT,Reaction Time;SD, Standard Deviation; VABS II, Vineland Adaptive Behaviour Scale Second Edition. | | | | | | | | | | | |  |
|  |  |  |  |  |  |  |  |  |  |  |  |  |

**Table S2. Outcome measures for 0-8 weeks monotherapy.**

| Endpoints | | | Lovastatin group (n=11) | | |  | Minocycline group (n=11) | | |  |
| --- | --- | --- | --- | --- | --- | --- | --- | --- | --- | --- |
|  |  |  | Baseline | 8 weeks | p |  | Baseline | 8 weeks | p |  |
| ABC-C_FX_ | | |  |  |  |  |  |  |  |  |
|  | Global score | | 48 (35-58) | 34 (31-51) | 0.350 |  | 42.5 (29-65) | 32.5 (16-50) | 0.100 |  |
|  | Irritability | | 8 (3-10) | 4 (3-7) | 0.136 |  | 9.5 (4-14) | 5 (2-8) | 0.261 |  |
|  | Lethargy | | 8 (6-14) | 9 (4-14) | 0.276 |  | 10.5 (5-14) | 6 (2-11) | 0.136 |  |
|  | Stereotypy | | 6 (4-10) | 5 (4-7) | 1 |  | 5 (4-6) | 5 (0-8) | 0.079 |  |
|  | Hyperactivity | | 7 (4-9) | 4 (3-7) | 0.105 |  | 7.5 (5-10) | 5 (2-9) | 0.124 |  |
|  | Inappropriate speech | | 5 (4-7) | 6 (4-8) | 0.492 |  | 7.5 (5-8) | 6 (4-7) | **0.009** |  |
|  | Social avoidance | |  |  |  |  |  |  |  |  |
| ADAMS | | |  |  |  |  |  |  |  |  |
| Total score | | | 28 (17-40) | 28 (15-33) | 0.876 |  | 30 (14-49) | 16 (12-36) | 0.072 |  |
|  | Manic/hyperactive behaviour | | 3 (1-4) | 2 (1-4) | 0.892 |  | 2 (1-8) | 3.5 (1-5) | 0.518 |  |
|  | Depressed mood | | 11 (8-15) | 13 (6-15) | 0.929 |  | 6.5 (5-11) | 5.5 (3-9) | **0.022** |  |
|  | Social avoidance | | 5 (3-13) | 8 (4-10) | 0.854 |  | 7.5 (4-10) | 6 (0.2-10) | 0.094 |  |
|  | General anxiety | | 2 (2-3) | 2 (2-4) | 0.188 |  | 4 (1-5) | 2 (1-3) | 0.257 |  |
|  | Obsessive/compulsive behaviour | |  | 4 (4-4) | 1 |  |  | 4 (3-4) | 0.174 |  |
| KiTAP | | |  |  |  |  |  |  |  |  |
|  | Alertness | |  |  |  |  |  |  |  |  |
|  |  | RT median | 417 (402-470) | 543 (537-547) | 0.500 |  | 380 (362-542) | 455 (384-618) | 0.500 |  |
|  |  | RT standard deviation | 143 (127-412) | 279 (260-329) | 1 |  | 201.5 (191-307) | 129 (121-317) | 1 |  |
|  | Distractibility | |  |  |  |  |  |  |  |  |
|  |  | Omissions with distractor | 3 (2-4) | 3 (1-8) | 0.089 |  | 2 (2-2) | 2 (0-5) | 0.089 |  |
|  |  | Omissions without distractor | 1 (0-2) | 1 (0-4) | 0.302 |  | 1 (0-3) | 0.5 (0-4) | 0.302 |  |
|  |  | Total omissions | 4 (3-6) | 5 (3-11) | 0.139 |  | 3 (2-5) | 2.5 (0-8) | 0.139 |  |
|  |  | Errors with distractor | 11 (6-14) | 5 (2-12) | **0.041** |  | 10 (8-12) | 5 (2-12) | **0.041** |  |
|  |  | Errors without distractor | 15 (2-17) | 4 (2-14) | 0.353 |  | 8 (2-14) | 3.5 (2-12) | 0.353 |  |
|  |  | Total errors | 27 (8-31) | 9 (4-25) | 0.086 |  | 16 (12-25) | 10.5 (4-21) | 0.086 |  |
|  | Flexibility | |  |  |  |  |  |  |  |  |
|  |  | RT median | 741 (636-1069) | 818 (783-1162) | 0.937 |  | 841 (672-975) | 727.8 (668-818) | 0.937 |  |
|  |  | RT SD | 419 (260-541) | 281 (165-611) | 1 |  | 377 (280-843) | 248 (204-302) | 1 |  |
|  |  | Errors | 12 (11-14) | 12 (11-14) | 1 |  | 11 (10-11) | 12.5 (11-15) | 1 |  |
|  | Inhibition | |  |  |  |  |  |  |  |  |
|  |  | RT median | 487 (362-556) | 440 (361-567) | 0.383 |  | 467.5 (402-518) | 482 (410-572) | 0.383 |  |
|  |  | RT SD | 137 (110-185) | 129 (87-215) | 0.742 |  | 123 (107-190) | 149 (69-178) | 0.742 |  |
|  |  | Errors | 3 (2-4) | 2.5 (1-6) | 0.799 |  | 2 (1-5) | 2.5 (1-5) | 0.799 |  |
| SRS | | |  |  |  |  |  |  |  |  |
|  | Total raw score | | 163 (157-170) | 146 (143-163) | **0.003** |  | 158.5 (132-176) | 148 (132-160) | 0.100 |  |
|  | Awareness | | 19 (18-21) | 17 (16-20) | 0.202 |  | 19.5 (18-21) | 18 (16-20) | 0.234 |  |
|  | Cognition | | 30 (27-33) | 31 (27-34) | 0.833 |  | 31.5 (26-33) | 30.5 (27-33) | 0.715 |  |
|  | Communication | | 52 (50-58) | 49 (45-53) | **0.042** |  | 48 (42-54) | 44.5 (41-48) | 0.426 |  |
|  | Motivation | | 29 (27-31) | 27 (24-29) | 0.113 |  | 24.5 (22-29) | 23.5 (21-26) | 0.227 |  |
|  | Mannerisms | | 32 (28-34) | 29 (26-32) | **0.006** |  | 29.5 (24-35) | 28.5 (23-31) | 0.057 |  |
| Data are presented as median (interquartile range); ABC-C_FX_, Aberrant Behaviour Checklist-Community adapted for FXS; ADAMS, Anxiety, Depression and Mood Scale; KiTAP, Test of Attentional Performance for Children; RT, Reaction Time; SD; Standard Deviation; SRS, Social Responsiveness Scale. | | | | | | | | | |  |
|  |  |  |  |  |  |  |  |  |  |  |
